# The NADPH Link between the Renin Angiotensin System and the Antioxidant Mechanisms in Dopaminergic Neurons

**DOI:** 10.3390/antiox12101869

**Published:** 2023-10-16

**Authors:** Rafael Franco, Joan Serrano-Marín, Gemma Navarro, Rafael Rivas-Santisteban

**Affiliations:** 1 Department of Biochemistry and Molecular Biomedicine, School of Biology, Universitat de Barcelona, 08028 Barcelona, Spain; 2 CiberNed, Network Center for Neurodegenerative Diseases, Spanish National Health Institute Carlos III, 28029 Madrid, Spain; g.navarro@ub.edu; 3 School of Chemistry, Universitat de Barcelona, 08028 Barcelona, Spain; 4 Department of Biochemistry and Physiology, School of Pharmacy and Food Science, Universitat de Barcelona, 08028 Barcelona, Spain; 5 Institute of Neurosciences, Universitat de Barcelona, 08007 Barcelona, Spain; 6 Campus Bellaterra, Autonomous University of Barcelona, Cerdanyola del Vallés, 08193 Barcelona, Spain

**Keywords:** Parkinson’s disease, glucose-6-phosphate dehydrogenase, NADPH, substantia nigra, brain, angiotensin receptors, MAS receptors, glutathione, 6-phospho-gluconate dehydrogenase, neuromelanin

## Abstract

The renin angiotensin system (RAS) has several components including signaling peptides, enzymes, and membrane receptors. The effort in characterizing this system in the periphery has led to the approval of a class of antihypertensives. Much less is known about RAS in the central nervous system. The production of RAS peptides and the expression of several RAS enzymes and receptors in dopaminergic neurons of the substantia nigra has raised expectations in the therapy of Parkinson’s disease, a neurodegenerative condition characterized by lack of dopamine in the striatum, the motor control region of the mammalian brain. On the one hand, dopamine production requires reducing power. On the other hand, reducing power is required by mechanisms involved in REDOX homeostasis. This review focuses on the potential role of RAS in the regulation of neuronal/glial expression of glucose-6-phosphate dehydrogenase, which produces the NADPH required for dopamine synthesis and for reactive oxygen species (ROS) detoxification. It is known that transgenic expression of the gene coding for glucose-6-phosphate dehydrogenase prevents the death of dopaminergic nigral neurons. Signaling via angiotensin II G protein-coupled receptors, AT_1_ or AT_2_, leads to the activation of protein kinase A and/or protein kinase C that in turn can regulate glucose-6- phosphate dehydrogenase activity, by Ser/Thr phosphorylation/dephosphorylation events. Long-term effects of AT_1_ or AT_2_ receptor activation may also impact on the concentration of the enzyme via activation of transcription factors that participate in the regulation of gene expression in neurons (or glia). Future research is needed to determine how the system can be pharmacologically manipulated to increase the availability of NADPH to neurons degenerating in Parkinson’s disease and to neuroprotective glia.

## 1. Introduction

**Figure 1:**
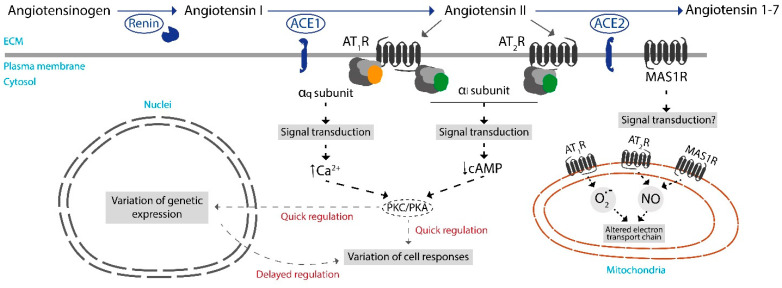
Main components of the renin angiotensin system (RAS). Receptors for angiotensin II have been described on the cell surface but also in mitochondrial membranes. Cell responses may be quick due to effector phosphorylation by PKA or PKC, or delayed, via regulation of gene expression and enhancement or repression of the synthesis of effector proteins. Details of angiotensin II and angiotensin 1-7 effects via mitochondrial receptors are detailed elsewhere [1,2]. NO = Nitric oxide. O_2_^•−^ = Superoxide anion.

The main components of the renin angiotensin system (RAS) include renin, angiotensinogen, angiotensin I, angiotensin II, angiotensin 1-7, angiotensin I converting enzyme 1 (ACE1), angiotensin converting enzyme 2 (ACE2), and several receptors (Figure 1). RAS has been extensively characterized in the periphery and in relation with renal function and the control of blood pressure. Less is known about the role of RAS in the central nervous system (CNS). An obvious function is the control of vascular tone because RAS members are expressed in endothelial and smooth muscle cells of brain vessels. However, RAS components are also expressed in cells of the CNS, neurons included. RAS participates in several ways in maintaining REDOX homeostasis in the brain. This review will focus on how RAS is involved in the production of NAPDH, which fuels the main antioxidant mechanisms in mammals.

Life on Earth evolved in an oxygen-rich atmosphere; a significant proportion of oxygen is used for biochemical reactions needed to sustain life on the planet. Mammals, as examples of higher organisms, have developed exquisite ways to use oxygen without burning themselves and to keep at bay the negative consequences of oxidation. Mammals fight against excess oxidation by absorbing the reduced molecules, mainly glucose/fructose and fatty acids, that are produced by plants from CO_2_ and the sun’s radiant energy. This review will focus on glucose, and more specifically on the lesser-known side of glucose, namely the role of glucose in providing the reducing power necessary to minimize oxidation in living cells; eukaryotic cells that consume oxygen produce oxidants as by-products and have developed efficacious antioxidant mechanisms.

We refer to our previous article in which we argued that “antioxidants” taken orally by mammals may not be able to effectively reduce oxidative stress [3]. Although there are different detoxification mechanisms depending on the tissue and environmental conditions, many of them depend on the production of NADPH. NADPH plays a crucial role in regenerating one of the most important component in antioxidant processes: reduced glutathione. There are several pathways that facilitate the regeneration of NADPH, of which the pentose phosphate pathway is the main one [4]. The two first reactions of the pathway, catalyzed by glucose-6-phosphate dehydrogenase (G6PDH) and 6-phospho-gluconate dehydrogenase (6PGNDH), produce NADPH. G6PDH is considered a key enzyme in the link between the pentose phosphate pathway and glycolysis. The pentose phosphate pathway is constituted by two phases, namely the oxidative phase, in which two NADPH molecules are produced for each glucose, and the non-oxidative phase, which is responsible for obtaining different types of pentoses that are used in anabolic processes. Even though NADPH is neither produced nor used in the non-oxidative phase, the overall NADPH production depends on the coordination of fluxes between the two (oxidative and non-oxidative) phases. Transaldolase 1, a product of the *TALDO1* gene, is the rate-limiting enzyme of the non-oxidative phase, that is, it is an important player in the regulation of the pathway.

Revisiting the role of this metabolic pathway, Stincone and colleagues highlighted in 2015 that the “(pentose phosphate pathway) function is critical to maintain redox balance under stress situations, when cells proliferate rapidly, in ageing, and for the ‘Warburg effect’ of cancer cells” [5]. The same review states that: “Therefore, the high neuronal sensitivity to mitochondrial dysfunction may be due to their inability to sustain elevated glycolysis because of their dependence on pentose phosphate pathway-based utilization of glucose” [5].

In the two main neurodegenerative diseases, Alzheimer’s disease (AD) and Parkinson’s disease (PD), it is becoming evident that mitochondrial alterations contribute to oxidative stress and neuronal death [6,7,8,9]. As depicted in Figure 1, angiotensin receptors are located on the cell surface, but they are also present in mitochondrial membranes [2,10,11], thus implying that RAS peptides have a direct role in regulating mitochondrial events. However, this review will not focus on antioxidant events that might be regulated by mitochondrial RAS components.

This review focuses on the management of oxidative stress based on NAPDH production in which RAS participates (i) by controlling vascular tone and, consequently, the availability of glucose by the brain and (ii) by regulating the expression and/or activity of proteins necessary for antioxidant actions in neurons through angiotensin and MAS1 proto-oncogene receptors expressed in neural cells. The description of pieces of the puzzle that are still missing in neurons and, more specifically, in nigral dopaminergic neurons where all RAS components are produced/expressed may guide future research aimed at improving our understanding of the RAS-antioxidant axis in the CNS.

## 2. Components of the RAS System

The most important RAS endogenous bioactive peptides are angiotensin II and angiotensin 1-7. They activate receptors that belong to the G protein-coupled receptor (GPCR) superfamily. According to the International Union of Pharmacology [12], there are two angiotensin II receptors, AT_1_ and AT_2_; both can couple to heterotrimeric G_i_ proteins, and their activation leads to inhibition of adenylate cyclase and the consequent decrease in the level of intracellular cAMP and inactivation of protein kinase A (PKA) (Figure 1). The AT_1_R can also couple to G_q_; in such a case, receptor activation leads to an increase in diacyl-glycerol and cytoplasmic calcium ion (Ca^2+^) and activation of protein kinase C (PKC) (Figure 1). It has been reported that angiotensin 1-7 activates the MAS1 receptor [13,14,15,16,17]. MAS1 was first identified in 1984 using a human epidermoid carcinoma cell line, and tumorigenic assays indicated that the isolated DNA corresponded to an oncogene. Immediately upon discovery, it was apparent that the protein product contained transmembrane domains and could be a GPCR [18]. Four years later, in 1988, MAS1 was postulated as an angiotensin receptor based on sequence similarity with the receptor of another peptide, neurokinin A, formerly known as substance K [19]. MAS1 is still considered an orphan receptor, probably due to doubts about whether a direct interaction between angiotensin 1-7 and MAS1 as reported in a heterologous expression system [20] is possible in natural cells. Another receptor that is potentially mediating the effect of RAS peptides is MAS-related G-protein coupled receptor member D (MRGPRD); in 2023, IUPHAR still considers it an orphan receptor (https://www.guidetopharmacology.org/GRAC/FamilyDisplayForward?familyId=16; accessed on 12 August 2023). 

Signal transduction through GPCRs has been extensively studied (Figure 1). Signaling begins with the cytosolic variation in the level of second messengers, mainly cAMP and Ca^2+^, that in turns leads to regulating the catalytic activity of protein kinases, mainly PKA and PKC. In this context, activation of protein kinases leads to the phosphorylation of effector proteins that shape the cell response. The effect of GPCR activation has two temporal components, one quick, within seconds of the interaction between, in this case, angiotensin II and AT_1_ or AT_2_ receptors, and another slow. The slow one, which takes several hours, is due to angiotensin II receptor-mediated regulation of transcription factors that enter the nucleus, leading to the regulation of gene transcription and, ultimately, to changes in the level of some specific cell proteins [12,21,22]. In the pharmacology of GPCRs, a pending issue is how their activation by agonists impacts on the regulation of the activity of metabolic enzymes and of enzymes related to redox homeostasis. For instance, does activation of RAS receptors lead to the regulation of G6PDH activity? Does it happen quickly, for instance by phosphorylating/dephosphorylating metabolic enzymes? Does it do it over the medium term by varying the concentration of the metabolic enzymes?

## 3. RAS in the CNS

Almost three decades ago, the density of angiotensin II AT_1_ and AT_2_ receptors was determined by radioligand binding in different brain areas of controls and of parkinsonian and dementia patients. Both receptors were found in all areas, and with two main conclusions: (i) the two receptors are expressed in neurons that degenerate in Parkinson’s disease (PD), i.e., in nigral dopaminergic neurons, and (ii) the levels of AT_2_ receptors in samples from patients with Alzheimer’s disease (AD) are increased in the temporal cortex [23].

RAS is important in several, if not all, types of neurons. For example, in a human cortical neuron cell line, the presence of angiotensin II was found to be associated with elevated levels of ROS and the formation of neurotoxic p-tau aggregates. This effect was reversed by AT_1_ receptor antagonists such as losartan [24]. Cortical neurons show a high rate of functioning of the pentose phosphate pathway, which probably compensates, among others, the pro-oxidant effect of angiotensin II thus constituting a defense mechanism against oxidative stress [25]. Dopaminergic neurons exhibit characteristics that increase their susceptibility to alterations in cellular antioxidant systems. This vulnerability arises mainly from the generation of ROS during dopamine metabolism [26], their elevated mitochondrial activity [27], and their propensity to accumulate heavy metals such as iron, which is a catalyst for the production of ROS [28].

It should be noted that the susceptibility of dopaminergic neurons to oxidative stress in humans is higher than in other mammals and lower species. Dopamine tends to oxidize and become aminochrome, which after tautomerization and polymerization gives rise to neuromelanin. Neuromelanin is very abundant in the human substantia nigra and this is the reason this human brain region macroscopically appears as dark. Furthermore, humans are the only animals that develop PD naturally [29,30]. The discovery of RAS components in dopaminergic pathways has raised hopes for antiparkinsonian actions by drugs targeting RAS. RAS regulates dopamine production and dopaminergic neurotransmission [31,32,33]. Does RAS also participate in the control of oxidative stress in neurons producing dopamine or responding to dopamine? Does RAS regulate NADPH production by affecting the activity and/or level of G6PDH? 

RAS component expression is not restricted to neurons because several components are expressed in glial cells. In diseases such as PD, that affect dopaminergic neurons, it is likely that microglial RAS impacts on neuroinflammation and oxidative stress (see [34] for review). RAS participates in the polarization of activated microglia; indeed, the expression of the proinflammatory or the neuroprotective phenotype is affected by angiotensin II and by the expression of RAS enzymes and receptors [1,35,36]. On the one hand, it is not well understood how RAS affects glucose metabolism in the brain, particularly the astrocyte-neuron lactate shunt [37]. On the other hand, mixed midbrain neuron-glia cultures treated with lipopolysaccharide to activate microglia leads to an increase in the expression of G6PDH [38]. Accordingly, it would be instrumental to decipher mechanisms of pentose phosphate pathway (PPP) regulation in neurons and glia by RAS and whether such potential regulation correlates with microglial polarization. Knowing how angiotensin II can affect PPP regulation may provide an answer on how to pharmacologically modulate RAS to prevent dopaminergic neuronal death by skewing microglia toward the neuroprotective phenotype.

Last but not least is the fact that glycogen is present in the glia and is mobilized when necessary [39]. Glycogen stores are necessary to supply glucose to neurons, obtain energy through glycolysis, and obtain NADPH through the PPP. Is RAS involved in regulating glucose and glycogen metabolism in the brain? Despite the lack of data, it is a plausible possibility.

## 4. NADPH Is Needed for Keeping REDOX Homeostasis in the CNS

### 4.1. Transgenic Expression of the Gene Coding for Glucose-6-Phosphate Dehydrogenase Prevents Nigral Neurodegeneration

The sensitivity of neurons to REDOX imbalance and, consequently, their dependence on the use of glucose by the pentose phosphate pathway, was raised decades ago [40]. Already in 1969, a report provided indirect evidence (in rat brain) that inhibition of the pentose pathway leads to neurological alterations [41]. This would fit with the data from a transcriptomics study in a mouse model of traumatic brain injury; the lesion leads to a strong increase in the expression of genes for three relevant enzymes in the pentose phosphate pathway, G6PDH, 6PGNDH, and transaldolase 1. Upon treatment with candesartan, an AT_1_ receptor (AT_1_R) antagonist with benefits in this specific traumatic brain injury model, the expression of the genes for both G6PDH and 6PGNDH did not change, suggesting that the injury-induced increase in the expression of these genes (measured in the hippocampus) is protective [42].

The potential of NADPH to prevent nigral neurodegeneration in PD was demonstrated in pioneering experiments on transgenic expression of the G6PDH in dopaminergic neurons of the substantia nigra; such an increase in activity led to neuroprotection in the 1-methyl-4-phenyl-1,2,3,6-tetrahydropyridine (MPTP) model of PD [43]. A subsequent study demonstrated that differentially expressed proteins upon transgenic expression of G6PDH were involved in detoxification and antioxidant defense [44]. In summary, neurons use glucose for the production of NADPH through the reaction catalyzed by G6PDH, whose greater activity in striatal dopaminergic neurons is neuroprotective. 

### 4.2. Role of the Pentose Phosphate Pathway in the CNS: Evidence from Patient Samples and from Preclinical Studies in Animal Models of Neurodegeneration

**Figure 2:**
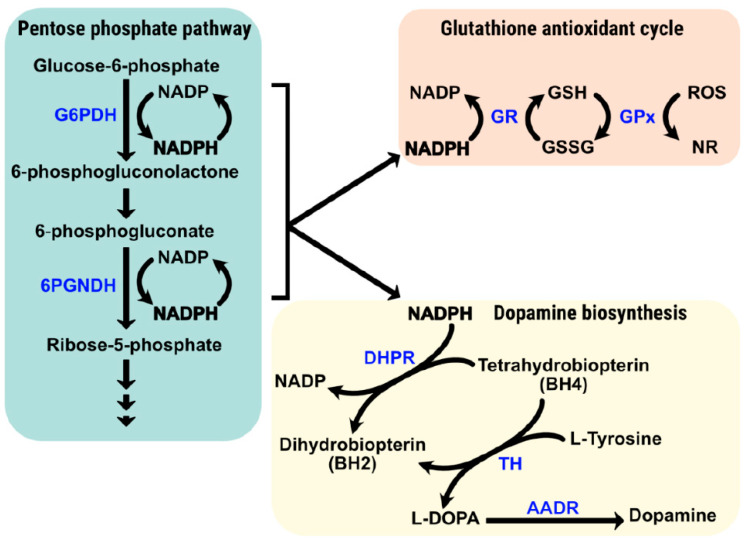
NADPH production and its participation in dopamine biosynthesis and glutathione-mediated antioxidant actions. Abbreviations: 6PGNDH = 6-phosphogluconate dehydrogenase. AADR = aromatic L-amino acid decarboxylase. DHPR = Dihydropteridine reductase. G6PDH = Glucose-6-phosphate dehydrogenase. GPx = Glutathione peroxidase. GR = Glutathione reductase. GSH = Reduced glutathione. GSSG = Oxidized glutathione. ROS = Reactive oxygen species. NR= Neutralized ROS (Non-reactive species/reduced species). TH = Tyrosine hydroxylase.

The pentose phosphate pathway is very active in neurons but also in glial cells. The production of NADPH by the oxidative branch of the pathway is needed for redox homeostasis in a pro-oxidant environment [45]. In fact, although it makes up a small percentage of the total body weight, oxygen consumption of the brain is estimated to be >20% of the human body’s requirements, with surges of up to 40% [46,47,48]. NADPH is co-substrate of the enzyme that produces the reduced form of glutathione, glutathione reductase (GSH). Reduced GSH is used to inactivate detrimental products such as reactive oxidative species by conversion into the oxidized form (GSSG) by the action of glutathione peroxidase [49]. This important antioxidant defense mechanism, which links glucose consumption with NADPH production and glutathione oxidation-reduction cycles (Figure 2), is present in neurons, astrocytes, and microglia [40,50,51]. 

The causes of the two main neurodegenerative diseases, PD and AD/dementia, can be genetic or idiopathic. In both cases, the underlying pathophysiological mechanisms, being heterogeneous, share altered mitochondrial function and/or production of oxidative stress. Mitochondrial dysfunction often correlates with increased oxidative stress. ROS are always produced as a byproduct of transfer of electrons from the NADH produced in the mitochondrial Krebs cycle to oxygen. The level of ROS production increases if there is a malfunction of the mitochondria and/or if the detoxification mechanisms are faulty or short of reducing power. Ultimately, shortage of reducing power means shortage of NADPH. 

In 1992, it was hypothesized that the higher vulnerability of catecholaminergic neurons in PD was due to “hyperoxidation” [52]. Dopamine is easily oxidized; the standard redox potential of the dopamine/dopamine quinone pair is fairly high (0.63 V; [53]). Production of aminochrome from dopamine correlates with oxidative stress in dopaminergic neurons [54]. Segura-Aguilar et al. indicated that the in situ oxidation of dopamine produces: “(i) mitochondria dysfunction, (ii) formation and stabilization of neurotoxic protofibrils of alpha synuclein, (iii) protein degradation dysfunction of both proteasomal and lysosomal systems and (iv) oxidative stress” [55]. Tetrahydrobiopterin, which is produced in catecholaminergic neurons, has been considered a triggering factor for PD. It can cause degeneration of dopaminergic terminals, i.e., nigral neuronal death, by production of reactive oxygen species. In vitro assays show that toxicity occurs in catecholaminergic cell lines but not in non-catecholaminergic ones. Importantly, the toxicity in sensitive cells markedly decreases using reduced (thiol) compounds such as GSH, *N*-acetylcysteine, β-mercaptoethanol and dithiothreitol [56]. Enzymes that participate in detoxification mechanisms also prevent the detrimental effect of tetrahydrobiopterin [56]. These in vitro findings fit with the marked decrease in nigral reduced glutathione [57] and with the expression of a GPX4 glutathione peroxidase in the substantia nigra of PD patients. Remarkably, the relative expression increases in surviving neurons. Those results are interpreted as if upregulation of GPX4, which uses NADPH as cosubstrate, is protective [58]. Measured as catalytic activity, glutathione peroxidase is decreased in homogenates from different brain regions, including the substantia nigra, of PD patients [59]. 

Infection of cells with a lentiviral vector carrying the sequence of human GPX1 glutathione peroxidase protects against the toxic effects of 6-hydroxydopamine. In addition, infection of nigral neurons using the same GPX1-gene-containing vector led to small but significant protection against intrastriatal injection of 6-hydroxydopamine [60]. The benefits of expression of the human glutathione peroxidase gene were also demonstrated in transgenic mice administered 6-hydroxydopamine intracerebroventricularly, where the authors concluded that “overexpression of glutathione peroxidase therefore partially protects dopaminergic neurons against 6-hydroxydopamine-induced toxicity” [61].

The increases in plasma and brain levels of angiotensin converting enzyme 1 (ACE1) that accompany cerebral ischemic stroke lesion in rats correlates with a marked decrease in the activity of catalase, superoxide dismutase, and glutathione peroxidase. Studies with oleuropein, a phenolic compound of olive *Olea europaea,* showed that the compound reduced brain edema and oxidative stress together with inhibiting ACE activity [62]. Correlation between expression of the gene for ACE1 and that of glutathione peroxidase in the hippocampus was shown in a rat model of vascular dementia when comparing animals treated with vehicle or with marinoid J, a complex phenylglycoside [63].

## 5. Peripheral RAS and Glucose Availability to the Brain

Blood is the main source of glucose for the brain, so any shortage of cerebral blood flow affects the availability of glucose to neural cells. From the point of view of energy production, neurons may depend in part on the glucose–lactate shunt by which astrocytes provide lactate to neurons. However, NAPDH-dependent detoxification mechanisms taking place in neurons require glucose in neurons. The involvement of the peripheral RAS in the control of cerebral blood flow cannot be easily assessed, but there are data to suggest that hypertension can lead to reduced blood flow to the brain. There are two possibilities to explain this correlation: it may be a consequence of hypertension, or rather, of antihypertensive medication, which often involves drugs that affect the RAS.

Blood glucose levels in healthy individuals increase with age [64]. The tendency to hypertension in aged individuals contributes to keeping the glucose availability to neurons. The position of our head in the upper part of the body makes it difficult for blood to rise from the heart to the brain. Also, the reduction with age of the flexibility of the blood vessels can reduce the blood flow that reaches the brain [65]. The less blood flow, the less availability of glucose in the brain. Upon these premises, RAS-targeted drugs used to combat hypertension may indirectly affect glucose availability in the brain. Despite the few studies and the technical challenge of assessing brain blood flow in humans, the prevalent view is that anti-hypertensives do not affect the cerebral blood flow [66]. In 2014, a report using MRI techniques in volunteers over 50 years of age showed that hypertension correlates with hypoperfusion of the cerebral cortex. It should be noted that 51.7% of the individuals in the sample were taking antihypertensive agents, mainly ACE1 inhibitors [67]. Although longitudinal studies are required to know the real effect of antihypertensives, it is tempting to speculate that chronically taken, anti-hypertensive drugs may lead to periods of hypotension which, in the long term and depending on range, frequency and duration, may affect glucose availability due to reduced blood flow to the brain [6]. In our opinion, this possibility highlights the relevance of comparing the long-term effects of drugs targeting RAS that differ in terms of their penetration into the brain. We suggest longitudinal studies assessing the onset of neurodegenerative diseases in hypertensive patients taking medication that does or does not cross the blood–brain barrier.

## 6. Regulation by RAS of the Expression of Genes Coding for Protein Needed in Antioxidant Routes 

### 6.1. Effect of RAS-Targeted Anti-Hypertensive Drugs 

Glucose is readily available in the periphery where RAS participates in minimizing oxidative stress. In the absence of any pathology, RAS is one of the many factors contributing to homeostasis. How may GPCR activation by RAS peptides lead to regulate REDOX events and impact detoxification mechanisms? The simplest explanation consists of regulating the activity of (i) enzymes that participate in detoxification mechanisms and (ii) enzymes that produce NADPH from glucose via the pentose phosphate pathway.

Several years ago, RAS-targeted drugs were approved to treat human hypertension (and some kidney diseases). The anti-antihypertensive effect of these drugs consists of (i) preventing the formation of angiotensin II (ACE1 inhibitors) or (ii) antagonizing the effect of angiotensin II on the AT_1_R (e.g., sartans) [68,69,70,71]. To our knowledge, there is no research aimed at addressing how angiotensin II affects the activity and/or the levels of G6PDH. Indirect evidence comes from significant effects on the heart levels of G6PDH upon administration of candesartan to a rat model of diabetes-induced oxidative damage [72]. Deficiency of G6PDH affects the angiotensin II effects on the blood pressure and decreased signaling towards AKT in isolated smooth muscle cells [73]. It is intriguing that these results were interpreted as if NADPH is mainly used for oxidative purposes via NAPDH oxidase. NADPH oxidase activity is essential for the functionality of phagocytes of the innate immune system, and its deficiency produces, primarily, chronic granulomatous disease and, secondarily, immunological response alterations that can include susceptibility to mycobacterial or pyogenic infections [74]. In addition, chronic granulomatous disease caused by deficiency of NADPH oxidase may lead to multiple clinical manifestations in virtually all tissues/organs, from the liver to the CNS (see [75] for review). This evidence goes against NAPH oxidase being harmful due to reactive oxygen species (ROS) production. In fact, NADPH oxidase was first described as a membrane-bound enzyme that allows transfer of electrons from NADPH to molecular oxygen for the synthesis of thyroid hormones [76,77,78]. In summary, NADPH oxidases are essential, and the most reliable studies show that they do not produce oxidative stress by default. Irrespective of whether some NADPH may be spent in producing ROS, it turns out that NADPH is necessary for the biosynthesis of dopamine in dopaminergic neurons and, in addition, there is consensus in that NADPH production is needed by several mechanisms of management of oxidative stress (Figure 2). Consistent with this view, reduced oxidative stress in diabetes-induced nephropathy correlates with enhanced activity of G6PDH [79]. Mutations that cause the loss of function of transaldolase 1, exacerbates oxidative stress due to an unbalanced pentose phosphate pathway and reduced NADPH availability [80]. Mutations in the transaldolase 1 (*TALDO1*) gene are rarely detected in adulthood, and symptoms have only been adequately described in newborns. Both in neonates inheriting the mutations and in *TALDO1* KO transgenic mice, symptoms greatly improve after antioxidant therapy [81,82]. 

### 6.2. Evidence of Angiotensin II Effects on AT_1_ and AT_2_ Receptors Using AT_1_R KO Mice 

Relevant information on how RAS can affect gene expression was obtained by a transcriptomic study in angiotensin II-treated wild-type and AT_1_R KO mice. Results using mRNA isolated from kidney allowed identifying differential effects of angiotensin II through AT_1_ and AT_2_ receptors. RAS is directly involved in the regulation of the expression of several components of the glutathione-based detoxification pathway. Nevertheless, it is probable that the activation of the AT_1_R is beneficial or detrimental depending on each specific scenario. On the one hand, AT_1_R activation leads to a reduction in the transcription of the glutathione synthase, but also increases the expression of the GPX6 glutathione peroxidase gene. On the other hand, AT_2_R activation by angiotensin II reduces the transcription of the 6PGNDH gene, of the glutathione reductase gene and of several glutathione S-transferase genes. The results also suggest that AT_1_R activation leads to a deficiency of NADP (and consequently of NADPH) because the genes for NAD synthase and NAD kinase (which converts NAD to NADP) are downregulated. In summary, in the kidney, (i) there is a direct link between the RAS and NADPH production and (ii) RAS contributes to the regulation of enzymes involved in glutathione-based detoxification mechanisms [83]. To our knowledge, no studies have used these transgenic animals to assess the effect of angiotensin II on the expression of genes in neurons and glia.

### 6.3. Expression of G6PDH in Cells Isolated from Patients with Preeclampsia 

RAS components increase during pregnancy, but the overall system remains balanced. However, the RAS is unbalanced in preeclampsia, a unique disease that occurs during pregnancy in which the kidney is primarily affected. Preeclampsia, which leads to high blood pressure in the mother, is life-threatening for both mother and fetus. In this disease, anti-AT_1_R antibodies appear in the serum, and there are possibilities that uteroplacental-produced angiotensin II can enter the systemic circulation [84,85]. A seminal study by Afzal-Ahmed et al. provided relevant information concerning the link between RAS and G6PDH activity, NADPH production, and redox status [86]. On the one hand, hexokinase, the activity of the enzyme that is required for glucose-6-P production, and G6PDH activity were significantly reduced (versus normotensive controls) in the erythrocytes of patients. Human umbilical vein endothelial cells (HUVEC) from normal-term and preeclamptic deliveries were also isolated, and the result was a significant decrease in G6PDH but not hexokinase in cells obtained from preeclamptic deliveries. On the other hand, the angiotensin II-induced production of O_2_^•−^ by HUVEC cells was completely abrogated in cells from patients [86]. Overall, the alterations in the RAS system found in preeclampsia include reduction of PPP flux, decrease in NADPH production, and altered angiotensin-II-mediated regulation of oxidative stress in cells obtained from patients.

## 7. Assessing the Potential of RAS to Regulate NADPH Production

**Figure 3:**
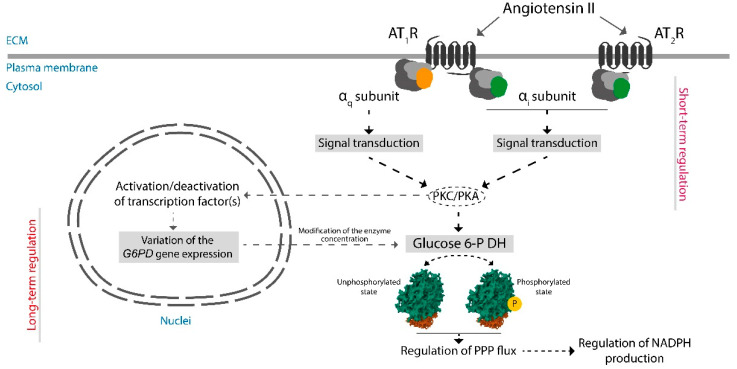
Possibilities for short- and long-term regulation of the production of glucose-6-phospha by G6PDH. Upon activation of AT_1_ or AT_2_ receptors, PKA and/or PKC may be involved in regulating the activity of the enzyme by phosphorylation/dephosphorylation events. Angiotensin II receptor-mediated signaling can lead, through activation/deactivation of transcription factors and over time, to regulate the expression of the *G6PDH* gene, leading to increases or decreases in the concentration of the enzyme. PPP: Pentose phosphate pathway.

As briefly commented earlier, there is very little information of the effect of GCPR activation on the activity of metabolic enzymes. The actual scenario contrasts with the pioneering studies showing that glucagon and catecholamines, via GPCR activation and changes in the level of intracellular cAMP, were regulating the activity of enzymes of glycogen metabolism [87,88,89,90,91,92]. The molecular mechanisms included PKA and involved phosphorylation/dephosphorylation of metabolic enzymes. Similar studies are required to know whether RAS impacts the activity of the enzymes of the pentose phosphate pathway. As activation of angiotensin II receptors leads to variation in the level of second messengers that in turn affect the activity of PKA and/or PKC (Figure 1), it is hypothesized that RAS receptor activation may regulate the activity of metabolic enzymes by phosphorylation/dephosphorylations events. There is strong evidence that activation of PKA and/or PKC affects the activity of enzymes of the pentose phosphate pathway. In glomerular mesangial cells exposed to different glucose concentrations, angiotensin II receptor regulation correlated with PKC translocation and activation [93]. It is likely that, via PKA and/or PKC, G6PDH activity is regulated in neurons that are responsive to angiotensin II and express G_q_/G_i_-coupled AT_1_/AT_2_ receptors (Figure 3). 

Variation in cAMP levels in bovine aortic endothelial cells correlate with the activity of G6PDH. Increases in cAMP levels cause a decrease in G6PDH activity, whereas inhibition of PKA activity, which is something that would occur upon activation of G_i_-coupled angiotensin receptors, reverted such decrease in the activity of G6PDH [94]. Also, via PKA activation, diabetes, induced by streptozotocin in rats, causes inhibition of glucose-6-phosphate dehydrogenase and increases oxidative stress in rat kidney [95]. Complementary interesting data were provided by Dieni and Storey (2010), who showed that G6PDH from wood frog *Rana sylvatica* liver can be phosphorylated by PKA, by PKC, and by calmodulin-dependent kinase, and that the higher the phosphorylation state of G6PDH, the higher the affinity of glucose for the enzyme [96]. In agreement with these results, unspecific activation of PKC by phorbol esters increases G6PDH activity measured in bovine coronary artery homogenates, likely by phosphorylating Ser^210^ and/or The^266^ residues [97].

In summary, although nigral neurons have both PKA and PKC, to our knowledge no research has been aimed at addressing how activation of angiotensin II receptors regulate metabolic enzymes such as G6PDH or 6PGNDH. Any information on this topic would lead to a better understanding of the NADPH link between the RAS and antioxidant mechanisms in dopaminergic neurons.

## 8. Conclusions

NADPH is a common factor in mechanisms related to REDOX homeostasis. To better understand the role of RAS in the regulation of antioxidant actions in the central nervous system, the effect of angiotensin II on the activity of NADPH-producing and degrading enzymes should be investigated. Nigral dopaminergic neurons constitute a very attractive model because (i) all the RAS components are produced and/or expressed, (ii) NADPH is needed for dopamine biosynthesis, and (iii) the intracellular environment is pro-oxidative, leading to the accumulation of neuromelanin, a product of the oxidation of dopamine. In addition, a better understanding of antioxidant mechanism would benefit parkinsonian patients as nigral neurodegeneration in these patients are due, at least, in part, to production of ROS and pro-oxidant molecules. The finding that transgenic expression of G6PDH confers protection to dopaminergic neurons goes in the same direction as that presented here. An important question that sums up the whole scenario is: What pharmacological manipulation of RAS should lead to short-term activation of NADPH-producing enzymes and/or long-term transcription-mediated increase in the concentration of these enzymes?
